# The health equity in all policies (HEiAP) approach before and beyond the Covid-19 pandemic in the Italian context

**DOI:** 10.1186/s12939-020-01209-0

**Published:** 2020-06-08

**Authors:** R. Bucciardini, B. Contoli, P. De Castro, C. Donfrancesco, L. Falzano, R. Ferrelli, A. M. Giammarioli, B. Mattioli, E. Medda, V. Minardi, G. Minelli, L. Palmieri, R. Pasetto, E. Pizzi, S. Rossi, A. Venerosi

**Affiliations:** Istituto Superiore di Sanità, Viale Regina Elena 299, 00161 Roma, Italy

For years, the scientific community has tried to promote the approach of Health Equity in All Policies (HEiAP) to policy decision-makers without having received an effective response through concrete actions; that has been the case in most countries of the world. Now, Covid-19 pandemic has not only clarified how “health” is affecting all other sectors of society and vice-versa, but it is also forcing policy makers to make important decisions, placing the concept of HEiAP at the top of the political agenda [1–4].

In the last decades many declarations and documents were elaborated with the aim of integrating health considerations with policies concerning sectors other than health, among the others *The Alma-Ata Declaration of 1978, The Ottawa Charter for Health Promotion in 1986, The Tallinn Charter in 2008* and *Health 2020* [5–8]*.* In all these documents, it is a common opinion that health depends only partly on the availability of health services, while much is related to the role of other non-health sector policies (development and employment, poverty, environment, school and education, social protection and security).

During the Covid-19 pandemic, we are experimenting with one of the most important concepts underlying the HEiAP approach: *How policy decisions affect health and health systems, including the distribution of health and equity in health systems.* Despite the widespread literature that warned us about a possible pandemic, the health systems have not equipped in time to deal with this pandemic [9–11].

If we consider the Italian spread of the pandemic there is an important gap in its health system that should be noted. In Italy, healthcare is provided to all citizens and residents by a national health coverage [12]. In 2000, Italy’s healthcare system was regarded as the second best in the world (after France) by the World Health Organization (WHO) [13, 14]. In the last years, Italy’s healthcare system has undergone some important changes [15, 16]. One of these changes regards regions gaining a considerable autonomy in determining the macrostructure of their health systems. Moreover, Italian regions have experienced a significant trend to promote the private sector (e.g. in most northern regions, including Lombardy, the region most affected by the pandemic) [17–19]. Concurrently, public hospitals have been merged, closed or heavily downsized. In addition, there has been a growing lack of decentralization of care, which could instead be very helpful in reducing the workload for hospitals. The pandemic has shown how much our healthcare system still relies primarily on hospitals and how much the territorial health system needs to be strengthened. In fact, while managing the emergency, hospital care should have been assisted more by local assistance. Furthermore, the accessibility and functionality of local health services are extremely different among regions [9, 12]. These choices have led to a lack of harmonization of best practices and sharing of standardized clinical with a loss of central control by the Government. At the same time, there have been severe cuts to the health system funding and lack of replacement of health workers [20]. Because of these political decisions, the Italian health system was unprepared to face a pandemic. In this context, the vulnerable and fragile people are often the most affected ones. Most of the victims have been elderly people with pre-existing unhealthy conditions. Moreover, due to overcrowding of hospitals, people with mild symptoms are quarantined at home. In this scenario, people living in small apartments and having lower living standards find it difficult to be adequately isolated from other members of the family to prevent contagion. In the same way, the most fragile people including those who live with mental ill health and isolation can exacerbate existing health problems [21]. Furthermore, we are experiencing a dramatically sudden evolution in vulnerabilities in the era of Covid-19 [22]. These groups can change or increase because of the side effects of the Covid-19 pandemic, such as those related to the loss of work or income, which means reducing opportunities of wellbeing and increasing the risk of unhealthy behaviours associated with unemployment or the lack of material resources. The loss of work and/or income is one of the major consequences of Covid-19. The International Labour Organization estimates that around 436 million enterprises in the hardest-hit sectors worldwide are currently facing high risks of serious disruption [23]. In Italy, the Ministry of Economy estimates a loss of workplaces of 2.1% in 2020 [24].

Similarly, the negative consequences of choices made in other non-health sectors are emerging dramatically during the pandemic. In Italy, the closure of schools due to the pandemic has highlighted some important gaps and fragility of the education system. As we know, education level can be considered one of the main social determinants of health. Despite this fact, interventions such as providing all students with adequate equipment to be able to study, in particular a computer and an internet connection have not been implemented. The education sector has been severely impacted by the COVID-19 pandemic for the lack of a digital system as well. Many teachers, students and families are for the first time grappling with the task of conducting online classes and remote learning, in the confinement of their homes. During the Covid-19 pandemic, we are dramatically experiencing how these social inequalities are turning into health inequalities, at global level. It is true that 2019–2020 coronavirus pandemic was not predicted, at least not by policy makers, but it is now highlighting how the HEiAP approach has not been introduced into the educational sector (*providing all students with all the necessary tools to guarantee an adequate level of education for everyone is a fundamental prerequisite for assuring everyone the same protection against not getting sick or dying*) [25–28].

Another important aspect that is emerging is related to the social position. Indeed, people with a more fragile social position suffer from the devastating effects of a pandemic. Once again, the pandemic is highlighting how the social position can indirectly affect health. In fact, the most economically fragile people (caregivers, housekeepers, precarious workers and all those who do not have adequate socio-economic protection) are those that will most likely have serious repercussions in terms of health implications even in the long run [25, 29].

In this scenario, promoting a sustainable development model based on an integrated approach of the several dimensions of development, from environmental to social and economic ones, is at the basis of the 2030 Agenda for United Nations Sustainable Development Goals (SDGs) [30, 31]. It is also important to highlight, after the lesson learnt from the Covid-19 pandemic, that Goal 3 “Good Health and Well-Being” should be now placed at the center of the Agenda and other global health models founded on intersectoral actions should be thought, as already underlined for responding to the widespread environmental degradation [32]. If before the pandemic, the understanding and mapping of important interactions among goals, sometimes, seemed to represent a theoretical exercise, now concrete actions should be implemented. In this context, a key objective of HEiAP is to aid informed policy makers. When different policy sectors are in competition, it is crucial that policy decision-makers are informed about these matters, taking into consideration any kind of implications on health and equity, supported by scientific evidence, and expressed in a way that they can easily understand.

An important component of this process is to define a strategy for an action plan and establish an evaluation framework and monitoring mechanism. Specific tools, indicators and analyses providing evidence-based data on how public policies and interventions impact health-related outcomes and health systems, as well as the distribution of these impacts across various population groups, should be considered. The results should be used to provide information and evidence from a health policy perspective at government level.

In this context, collaboration between all health and non-health stakeholders is essential and it is also necessary to strengthen the role of the health sector with stewardship.

With the purpose of promoting the HEiAP approach and with the SDGs vision guiding its activity, the main Italian research institute for public health – Istituto Superiore di Sanità (ISS), Italian National Institute of Health, the technical and scientific body of the Italian National Health Service - has recently established a cross-disciplinary Unit involving researchers from different backgrounds [33]. Thanks to the ISS organization, including multidisciplinary expertise, the HEiAP approach has become part of its institutional Agenda with the main goal of promoting the inclusion of health considerations in all policies and advocating their effective implementation across sectors in order to maximize health benefits.

This commitment aims to guarantee scientific evidence able to support and drive policies and actions and improve Italy’s role within the European panorama in terms of HEiAP approach acting on SDGs.
